# Association between hypothyroidism and metabolic profile in gestational diabetes mellitus

**DOI:** 10.3389/fendo.2025.1614802

**Published:** 2025-09-25

**Authors:** Sara Pinto, Charlotte Nachtergaele, Laura Croce, Lionel Carbillon, Amélie Benbara, Emmanuelle Fabre, Mario Rotondi, Emmanuel Cosson

**Affiliations:** ^1^ Assistance Publique - Hôpitaux de Paris (AP-HP), Avicenne Hospital, Paris 13 University, Sorbonne Paris Cité, Department of Endocrinology-Diabetology-Nutrition, Centre de Recherche en Nutrition Humaine - Ile de France (CRNH-IdF), Centre Spécialisé de l’Obésite Île-de-France Nord, Bobigny, France; ^2^ Assistance Publique - Hôpitaux de Paris (AP-HP), Ambulatory Unit of Endocrinology-Diabetology-Nutrition, Jean Verdier Hospital, Université Paris 13, Sorbonne Paris Cité, Centre de Recherche en Nutrition Humaine - Ile de France (CRNH-IdF), Centre Speécialisé de l’Obeésite Ile-de-France Nord (CINFO), Bondy, France; ^3^ Assistance Publique - Hôpitaux de Paris (AP-HP), Unité de Recherche Clinique St-Louis-Lariboisière, Université Denis Diderot, Paris, France; ^4^ Department of Internal Medicine and Therapeutics, University of Pavia, Pavia, Italy; ^5^ Unit of Endocrinology and Metabolism, Laboratory for Endocrine Disruptors, Istituti Clinici Scientifci Maugeri Istituto di Ricovero e Cura a Carattere Scientifico (IRCCS), Pavia, Italy; ^6^ Assistance Publique - Hôpitaux de Paris (AP-HP), Jean Verdier Hospital, Paris 13 University, Sorbonne Paris Cité, Fédération Hospitalo-Universitaire “Early Identification of Individual Trajectories in Neuro-Developmental Disorders” (I2D2), Department of Perinatology and Gynecology, Bondy, France; ^7^ Assistance Publique - Hôpitaux de Paris (AP-HP), Avicenne and Jean Verdier Hospitals, Paris 13 University, Sorbonne Paris Cité, Biochemistry Department, Bobigny, France; ^8^ Université Sorbonne Paris Nord and Université Paris Cité, Institut National de la Santé et de la Recherche Médicale (INSERM), UMR-978 “Signalisation, Microenvironnement et Hémopathies Lymphoïdes” Université Sorbonne Paris Nord, Bobigny, France; ^9^ Université Sorbonne Paris Nord and Université Paris Cité, Institut National de la Santé et de la Recherche Médicale (INSERM), Institut National de Recherche Pour l’agriculture, l’alimentation et l’environnement (INRAE), Caisse Nationale Assurance Maladie (CNAM), Center of Research in Epidemiology and Statistics (CRESS), Nutritional Epidemiology Research Team (EREN), Bobigny, France

**Keywords:** gestational diabetes, hypothyroidism, TSH, thyroid, pregnancy

## Abstract

**Introduction:**

Thyroid hormones exert many effects on glucose metabolism. Gestational diabetes mellitus (GDM) and hypothyroidism during gestation (HG) are the most common gestational endocrinopathies and seem to be associated. We therefore explored in women with GDM whether the presence of HG is associated with a different metabolic profile.

**Materials and methods:**

We included 1,290 pregnant women with GDM [International Association of the Diabetes and Pregnancy Study Group (IADPSG)/World Health Organization (WHO) criteria] and no history of hypothyroidism prior to pregnancy who had a measure of thyroid-stimulating hormone (TSH) and anti-thyroperoxidase antibodies during their hospital stay after GDM diagnosis. Patients with thyrotoxicosis and previous bariatric surgery were excluded. We evaluated concomitant blood pressure, fasting glycemia, insulinemia [with calculation of homeostatic model assessment for insulin resistance (HOMA-IR) index], glycated hemoglobin (HbA1c), and lipid profile according to the presence of HG (American Thyroid Association 2017 definition: TSH ≥ 4 mUI/L).

**Results:**

The mean (± standard deviation) age was 33 ± 5 years, the mean body mass index was 27 ± 5 kg/m^2^, and 117 women (9%) displayed HG. HG was associated with higher HbA1c (5.35 ± 0.56% *vs*. 5.22 ± 0.52%, *p* = 0.009), even after adjustment for gestational age, age, and body mass index. TSH was also positively associated with HbA1c (*p* = 0.006) and HOMA-IR (*p* = 0.002). Patients with HG displayed less often an early GDM, with their fasting glycemia before 24 weeks of amenorrhea being lower than that of patients with a TSH < 4 mU/L.

**Conclusion:**

In our cohort of patients with GDM, women with HG showed higher HbA1c than those without and HOMA-IR was positively associated with the level of TSH.

## Introduction

Thyroid hormones are known to exert important effects on glucose homeostasis. These effects may be opposite according to the target organ, as they act as agonists of insulin in the muscle and as antagonists of insulin in the liver (1). Hypothyroidism has been shown to be associated with peripheral insulin resistance, which is characterized by reduced peripheral glucose utilization and, in addition, by a decrease in hepatic gluconeogenesis and glycogen synthesis (2).

In non-pregnant subjects, two studies reported an increased risk of type 2 diabetes in patients with hypothyroidism (3, 4). Furthermore, some studies have suggested that increasing thyroid-stimulating hormone (TSH) levels are associated with hyperglycemia and insulin resistance even in euthyroid patients (5, 6).

Hypothyroidism during gestation (HG) and gestational diabetes mellitus (GDM) are the most common endocrinopathies during pregnancy. Both conditions seem to be associated (7, 8). Moreover, having a TSH ≥4 mUI/L during pregnancy increases the risk of GDM independently from anti-thyroperoxidase antibodies (aTPO) status (9). The heightened risk may be attributed to the impact of hypothyroidism in exacerbating the physiologic gestational insulin resistance. It has been demonstrated that during the second half of pregnancy, the hormonal environment promotes a catabolic status in which there is a progressive increase in insulin resistance (10). In the presence of some pregestational conditions (i.e., obesity and advanced age), this insulin resistance may overcome the beta-cell capacity to increase insulin secretion and elicit a dysglycemic status, namely, GDM (10).

GDM was historically defined as any degree of glucose intolerance with an onset or first recognition during pregnancy. This definition has many limitations mainly because GDM is a heterogeneous condition.

According to the 2017 American Thyroid Association (ATA) guidelines on thyroid disease in pregnancy (11), an upper limit of normality (≈4.0 mUI/L for most TSH assays) should be used to diagnose HG in a pregnant patient. The presence or absence of positive tests for aTPO was suggested to be taken into account for treatment decision-making.

To the best of our knowledge, no studies have investigated the role of HG on glucose metabolism in women with GDM. The aim of our study was to correlate the presence of HG to metabolic parameters in a cohort of patients with GDM.

## Materials and methods

### Participant selection

The present retrospective, observational study was conducted at Jean Verdier University Hospital in a suburban area of Paris (Bondy), France. It was based on the electronic medical records of every woman who delivered between 1 January 2012 and 31 December 2018. Women were informed that their medical records could be used for research purposes unless they were opposed to such use; data were analyzed anonymously. Our database is registered in the French Committee for computerized data (Commission Nationale de l’Informatique et des Libertes, no. 1704392v0).

Exclusion criteria were no personal history of either pre-gestational diabetes or bariatric surgery and hypothyroidism. Inclusion criteria were the presence of GDM, age 18–50 years, singleton pregnancy, and measurement of TSH and aTPO during their hospital stay after GDM diagnosis. We then excluded the women with TSH level < 0.27 mUI/L.

Our policy was a universal screening of GDM at both the beginning of pregnancy and after 24 weeks of amenorrhea (WA) if previous screening either had been normal or had not been done. Early screening was based on fasting plasma glycemia (FPG) measurement, whereas late screening was based on a 75-g oral glucose tolerance test (OGTT) with measurement of fasting, 1-h, and 2-h plasma glucose levels. GDM was defined according to International Association of the Diabetes and Pregnancy Study Group (IADPSG)/World Health Organization (WHO) recommendations (12, 13), as these guidelines have been endorsed in France (14). We included both women with early fasting hyperglycemia (early-diagnosed GDM: FPG of 5.1–6.9 mmol/L before 24 WA) and patients with a pathological OGTT after 24 WA (FPG at 5.1–6.9 mmol/L and/or 1-h plasma glucose 10.0 mmol/L and/or 2-h plasma glucose at 8.4–11.0 mmol/L during an OGTT) (14). Note that overt diabetes was defined as FPG ≥ 7 mmol/L or HbA1c ≥ 6.5%. In our department, after the diagnosis of GDM, the patient is invited to spend 1 day at hospital (DH), where she meets a diabetologist, a dietician, and a nurse, and a blood sample is taken. Women with HG received their DH workup later as compared to women without HG (30.7 ± 5.0 *vs*. 28.4 ± 5.6 weeks, *p* ≤ 0.001), maybe because their screening after 24 WA was performed later too (27.8 ± 3.2 *vs*. 27.1 ± 3.1 WA, *p* = 0.025).

Blood pressures were measured after 10 min of resting.

Our local policy was a selective screening for HG according to ATA recommendations (15) at the first trimester, but first-trimester TSH values were not available in the dataset.

### Laboratory assays

The serum levels of TSH and serum titers of aTPO were measured using electrochemiluminescence immunometric assay dedicated for cobas^®^ e 601 analyzer (Elecsys TSH and aTPO assays, cobas^®^, Roche Diagnostics™, France). The sensitivity of the TSH and aTPO assays was 0.005 mIU/L and 5 IU/mL, respectively. According to TSH or aTPO levels, intra- and inter-assay coefficients of variation (CVs) reported by the manufacturer ranged from 1.3% to 11.1% and from 2.0% to 11.9% for the TSH assay, respectively. Intra- and inter-CV ranged from 2.8% to 4.8% and from 3.5% to 6.1% for the aTPO assay, respectively. Expected TSH serum levels range from 0.27 to 4.2 mUI/L. A borderline value of 34 IU/mL was defined for the aTPO assay.

Glucose values were measured on venous plasma using the enzymatic reference method with hexokinase (Cobas c 501 analyzer, Roche Diagnostics, France). Glycated hemoglobin (HbA1c) measurement was performed on hemolyzed whole blood using a turbidimetric inhibition immunoassay (c501 cobas^®^, Roche Diagnostics™, France).

The insulin level was measured in serum samples of some unselected women using the Roche Cobas electrochemiluminescence immunometric assay (Cobas e 601 analyzer, Roche Diagnostics, France). The intra-assay CV (repeatability) was 3.7% and the inter-assay CV (reproducibility) was 4.6%. The homeostatic model assessment for insulin resistance (HOMA-IR) index was calculated (16).

Total and high-density lipoprotein (HDL) cholesterol measurement was based on a colorimetric assay on the homogeneous phase and cholesterol dosage by cholesterol oxidase, measurement of triglycerides was based on a colorimetric assay, and low-density lipoprotein (LDL) cholesterol was calculated using the Friedewald formula. All these measurements were performed on plasma from fasting individuals using a Cobas 6000 analyzer (Roche Diagnostics, Meylan, France).

### Adverse pregnancy outcomes

Levothyroxine therapy was prescribed in accordance with the 2011 ATA guidelines (15) if a TSH >2.5 or >3 mIU/L was found during or after the first gestational trimester, respectively. Because women with HG were eventually treated, the analysis of pregnancy outcomes by HG status was only exploratory.

Insulin treatment was prescribed only if, after 2 weeks of diet and physical activity, pre-prandial and/or 2-h post-prandial glucose levels were >5.0 mmol/L and/or 6.7 mmol/L, respectively, ≥3 times/week, as recommended by French guidelines (14).

Definitions of pregnancy outcomes are provided in previous publications (17–21). Gestational weight gain was defined as the weight measured before delivery minus self-reported pre-pregnancy weight.

### Statistical analysis

Baseline continuous variables were expressed as mean ± standard deviation (SD). Categorical variables were expressed as frequencies (percentages). No data replacement procedure was used for missing data.

We analyzed the characteristics of the population according to the presence of HG defined as a TSH level >4 mU/L.

To compare the characteristics in the two groups (TSH ≤4 *vs*. >4 mUI/L), we used Student’s *t*-test or the Mann–Whitney test for Gaussian and non-Gaussian continuous variables, respectively, and chi-squared (*χ* ^2^) or Fisher’s exact test for categorical variables. We also evaluated TSH as a continuous variable and evaluated its association with metabolic parameters (FPG, HOMA-IR, and, in a subgroup of women, lipid profile and blood pressure) with linear regression. A multivariate linear model was designed including HbA1c and HOMA-IR as dependent variables and TSH (mUI/L), WA (weeks), BMI (kg/m^2^), and age (years) as covariates.

All tests were two-sided. Analyses were conducted using the R 3.6.3 software (R foundation, Vienna, Austria, https://cran.r-project.org).

## Results

### Women characteristics

**Figure 1:**
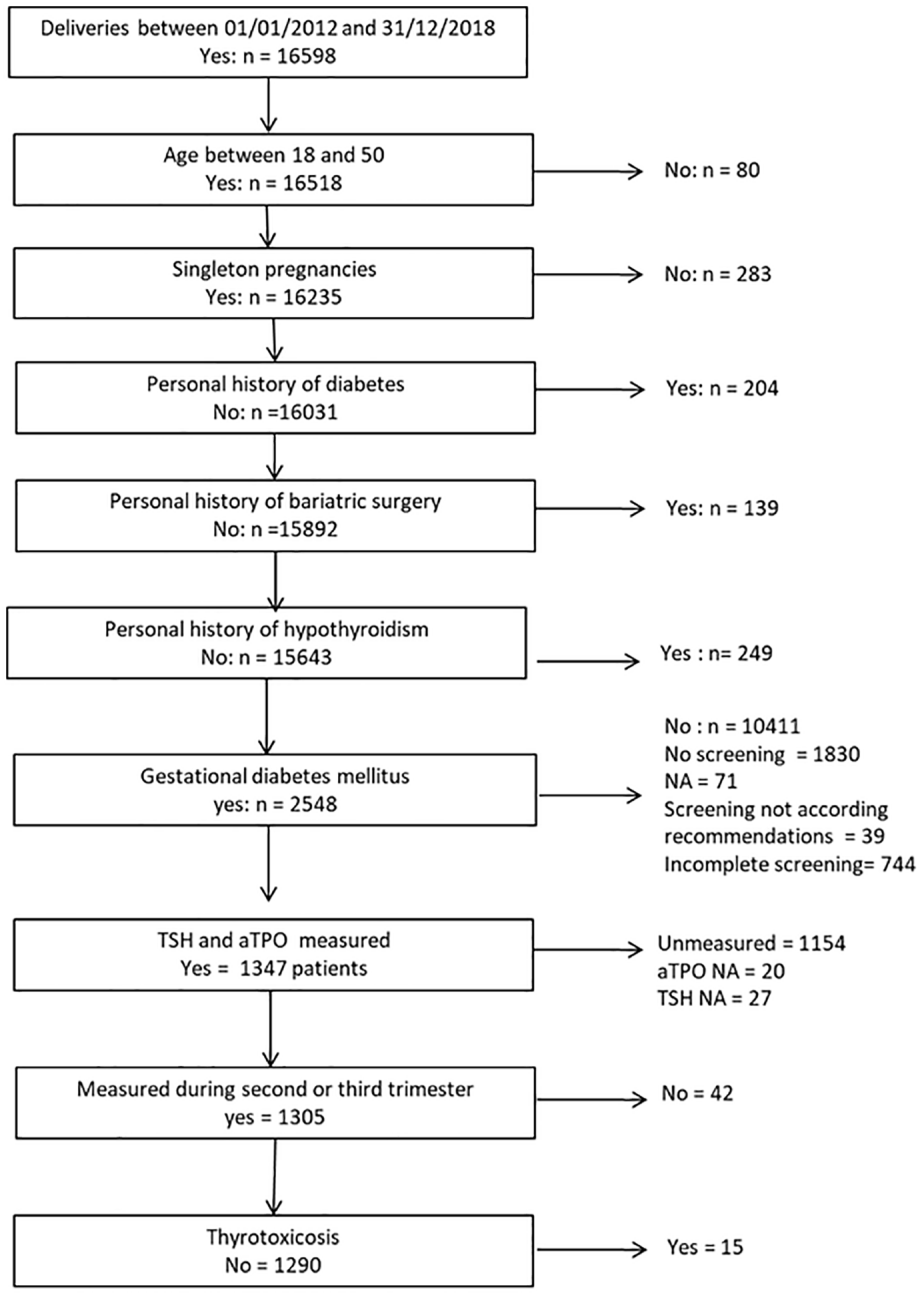
Flowchart of the study.

A total of 1,290 women (flowchart in **Figure 1**), 33 ± 5 years old, with a body mass index of 27 ± 6 kg/m², from multiple ethnicities were ultimately included in our observational study; their characteristics are shown in **Table 1**. Included patients had been admitted 1 day at hospital for education and care at 28.5 ± 5.6 WA, with a delay of 3.4 ± 3.3 weeks between GDM diagnosis and thyroid workup.

**Table 1:** Characteristics of population.

	Total (*N* = 1,290)	TSH [0.27–4.0] (*N* = 1,173)	TSH >4.0 (*N* = 117)	*P*
Age (years)	32.91 (5.40)	33.08 (5.31)	31.26 (5.98)	<0.001
Age (years), *n* (%)				<0.001
<30	354 (27.4%)	306 (26.1%)	48 (41.0%)	
≥30	936 (72.6%)	867 (73.9%)	69 (59.0%)	
BMI (kg/m^2^)	27.29 (5.65)	27.35 (5.66)	26.75 (5.55)	0.2873
Ethnicity				<0.001
Sub-Saharian Africa	217 (16.8%)	199 (17.0%)	18 (15.5%)	
North Africa	446 (34.6%)	413 (35.2%)	33 (28.4%)	
Other	98 (7.6%)	89 (7.6%)	9 (7.8%)	
Europe	239 (18.6%)	230 (19.6%)	9 (7.8%)	
Haiti, DOM/TOM	60 (4.7%)	50 (4.3%)	10 (8.6%)	
Pakistan, India, Sri Lanka	228 (17.7%)	191 (16.3%)	37 (31.9%)	
Missing	2	1	1	
Family history of diabetes, *n* (%)				0.5665
No	836 (64.8%)	763 (65.0%)	73 (62.4%)	
Yes	454 (35.2%)	410 (35.0%)	44 (37.6%)	
Personal history of GD, *n* (%)				0.6148
1st pregnancy	378 (29.3%)	325 (27.7%)	53 (45.3%)	
No	693 (53.7%)	651 (55.5%)	42 (35.9%)	
Yes	219 (17.0%)	197 (16.8%)	22 (18.8%)	
Personal history of macrosomia, *n* (%)				0.3642
1st pregnancy	378 (29.3%)	325 (27.7%)	53 (45.3%)	
No	846 (65.6%)	786 (67.0%)	60 (51.3%)	
Yes	66 (5.1%)	62 (5.3%)	4 (3.4%)	
Personal history of fetal loss, *n* (%)				0.0706
1st pregnancy	219 (17.0%)	196 (16.7%)	23 (19.7%)	
No	1030 (79.8%)	936 (79.8%)	94 (80.3%)	
Yes	41 (3.2%)	41 (3.5%)	0 (0.0%)	
Smoking before pregnancy, *n* (%)				0.8528
No	1185 (91.9%)	1077 (91.8%)	108 (92.3%)	
Yes	105 (8.1%)	96 (8.2%)	9 (7.7%)	
Smoking during pregnancy, *n* (%)				0.5306
No	1231 (95.4%)	1118 (95.3%)	113 (96.6%)	
Yes	59 (4.6%)	55 (4.7%)	4 (3.4%)	
Parity	2.37 (1.27)	2.40 (1.27)	2.06 (1.24)	0.006
Fetal sex, *n* (%)				0.2470
Female	628 (48.7%)	577 (49.2%)	51 (43.6%)	
Male	662 (51.3%)	596 (50.8%)	66 (56.4%)	
Diagnosis				0.017
Early GD	367 (28.4%)	347 (29.6%)	20 (17.1%)	
GD	835 (64.7%)	747 (63.7%)	88 (75.2%)	
Overt diabetes	88 (6.8%)	79 (6.7%)	9 (7.7%)	
WA of early screening *N* = 833	12.53 (5.77)	12.52 (5.90)	12.57 (4.27)	0.9312
Fasting glycemia (mmol/L) at early screening *N* = 807	5.17 (0.79)	5.19 (0.81)	4.97 (0.49)	0.0017
WA at OGTT *N* = 980	27.21 (3.10)	27.13 (3.08)	27.87 (3.20)	0.0247
Fasting glycemia (mmol/L) at OGTT *N* = 932	5.12 (0.73)	5.12 (0.73)	5.09 (0.78)	0.7055
1-hour glycemia (mmol/L) at OGTT *N* = 866	9.54 (2.01)	9.53 (1.99)	9.57 (2.17)	0.8665
2-hour glycemia (mmol/L) at OGTT *N* = 875	8.27 (1.99)	8.27 (2.00)	8.29 (1.93)	0.9255

BMI, body mass index; WA, week of amenorrhea; GD, gestational diabetes; OGTT, oral glucose tolerance test.

### Percentage of HG and parameters associated with HG

A total of 117 women (9%) displayed HG. **Table 1** shows that they were younger and with lower parity as compared to women without HG. Ethnicity also differed by HG status because of the higher prevalence of women from India, Pakistan, Sri Lanka, and Haiti or DOM/TOM.

**Table 1** also shows that women without HG more likely had an early-diagnosed GDM (29.6 *vs*. 17.1%, *p* = 0.017), and their FPG level before 22 WA was higher (5.2 ± 0.8 *vs*. 5.0 ± 0.5 mmol/L, *p* = 0.0017). Glucose profile at screening OGTT was similar in both groups.

### Correlation between TSH and metabolic parameters at DH

As shown in **Table 2**, women with HG had a positive aTPO more frequently (16.2% *vs*. 5.3%, *p* < 0.001) and displayed slightly higher HbA1c (5.35 ± 0.6% *vs*. 5.2 ± 0.5%, *p* = 0.0009), even after adjustment for WA at DH, age, ethnicity, and BMI, as they were younger (*p* = 0.0240). No differences were found in terms of HOMA-IR. In a subgroup of women for whom these variables were available, lipids and blood pressure levels were similar by HG status.

**Table 2:** Hospital stay parameters according to the presence of hypothyroidism during gestation.

	Total (*N* = 1,290)	TSH [0.27–4.0] (*N* = 1,173)	TSH > 4.0 (*N* = 117)	*P*
WA at hospital stay	28.58 (5.60)	28.37 (5.62)	30.67 (5.03)	<0.001
Delay between OGTT and DH (weeks)	3.38 (3.31)	3.32 (3.33)	3.86 (3.11)	0.1261
Hospital stay trimester				<0.001
T2	461 (35.7%)	437 (37.3%)	24 (20.5%)	
T3	829 (64.3%)	736 (62.7%)	93 (79.5%)	
TSH (mUI/L)	2.27 (1.26)	1.99 (0.86)	5.05 (1.31)	<0.001
aTPO				<0.001
Negative	1209 (93.7%)	1111 (94.7%)	98 (83.8%)	
Positive	81 (6.3%)	62 (5.3%)	19 (16.2%)	
LT4 (>2.5 mUI/L at T1, >3 mU/L at T2 or T3)				<0.001
No	994 (77.1%)	994 (84.7%)	0 (0.0%)	
Yes	296 (22.9%)	179 (15.3%)	117 (100.0%)	
Fasting glycemia (mmol/L) at hospital stay *N* = 1,288	4.63 (0.78)	4.64 (0.78)	4.57 (0.81)	0.3534
HbA1c at hospital stay *N* = 1,287	5.23 (0.53)	5.22 (0.52)	5.35 (0.56)	0.0090
Insulin (mUI/L) *N* = 1,268	14.66 (10.26)	14.53 (10.26)	15.90 (10.17)	0.1730
HOMA-IR *N* = 1,266	3.13 (2.71)	3.11 (2.70)	3.37 (2.74)	0.3244
HDL-c (mmol/L) *N* = 243	1.74 (0.40)	1.74 (0.40)	1.74 (0.42)	0.9236
Non-HDL-c (mmol/L) *N* = 242	4.08 (1.13)	4.04 (1.14)	4.33 (1.05)	0.1951
Triglycerides, mmol/L *N* = 243	2.19 (0.87)	2.18 (0.88)	2.21 (0.79)	0.8591
DBP (mmHg) *N* = 990	68.10 (9.84)	68.07 (9.92)	68.46 (9.07)	0.7123
SBP (mmHg) *N* = 994	111.74 (11.30)	111.67 (11.39)	112.39 (10.45)	0.5630

WA, week of amenorrhea; LT4, levothyroxine treatment; DBP, diastolic blood pressure; SBP, systolic blood pressure; OGTT, oral glucose tolerance test.

When considering TSH as a continuous variable, we found a positive correlation between TSH and HbA1c (*p* = 0.0058) and HOMA-IR (*p* = 0.002), even after adjustment for WA at DH, age, and BMI (*p* = 0.0240, and *p* = 0.002, respectively, **Table 3**).

**Table 3:** Linear regression analysis.

Linear regression analysis				
	TSH			
	Regression coefficient	95% CI		P
Dependent variables		Lower	Upper	
HbA1c	0.183	0.053	0.313	0.006
HOMA-IR	0.040	0.015	0.066	0.002
WA at hospital stay	0.037	0.025	0.049	<0.001
Age	−0.028	−0.036	−0.011	<0.001
BMI	0.0006	−0.012	0.013	0.928
Multiple regression analysis using HbA1c and HOMA-IR as dependent variables and TSH (mUI/L), WA (weeks), BMI (kg/m^2^), and age (years) as covariates.				
	HbA1c			
	Regression coefficient	95%CI		p
		Lower	Upper	
TSH	0.0262	0.0034	0.0489	0.0240
WA at hospital stay	0.0106	0.0055	0.0158	0.0001
Age	0.0070	0.0017	0.0123	0.0095
BMI	0.0192	0.0142	0.0242	<0.0001
	HOMA-IR			
	Regression coefficient	95% CI		p
		Lower	Upper	
TSH	0.1879	0.0690	0.3067	0.0020
WA at hospital stay	−0.0005	−0.0274	0.0264	0.9711
Age	0.0010	−0.0266	0.0287	0.9407
BMI	0.0987	0.0726	0.1249	<0.0001

WA, week of amenorrhea.

## Discussion

The present study evaluates the association between metabolic parameters and TSH considered as both categorical (cutoff, 4 mUI/L) and continuous variables in a cohort of women with GDM.

We found that women with HG displayed slightly higher HbA1c than those without and TSH levels were positively associated with HbA1c. These findings could be explained by a synergistic effect of HG and pre-gestational insulin resistance. Even if not associated with HG, HOMA-IR showed a correlation with increasing TSH without a cutoff. Only another study (22) explored HbA1c level in women with GDM according to the presence of euthyroidism or HG. It did not find any difference, maybe because the diagnosis of HG was made when TSH was ≥3 mUI/L and fT4 level was <0.76 ng/dL.

Together with the role of hypothyroidism in increasing peripheral insulin resistance, GH could promote the onset of GDM through an impairment of the placentation process (8). Indeed, the placenta is the main barrier between fetal and maternal environments and regulates fetal nutrition. Moreover, it has a central role in determining insulin resistance during pregnancy through its hormonal and cytokine secretion. Thyroid dysfunction and autoimmunity can cause alterations in the development of the feto-placental unit (23), as assessed by abnormalities in uterine artery pulsatility and in placental histology (23–25). Early-pregnancy hCG concentrations, which are reduced in abnormal placentation (26), are inversely related with GDM risk (27–29). These data suggest that placental abnormalities could be a possible physio-pathologic link between GH and GDM. In a small subgroup of women from our population where these parameters were available, no difference was found in terms of lipid and blood pressure levels. Indeed, a retrospective cohort study (30) evaluated the relationship between first-trimester thyroid function and lipid levels: as compared with the euthyroidism group, the hypothyroidism group (TSH > 3.52 mUI/L) had higher total cholesterol and LDL cholesterol levels; total cholesterol levels were positively correlated with TSH. The observed discrepancies between the former study and ours may be attributed to the varying gestational age when TSH measurement was performed.

In our study, women with HG were less likely to have an early-diagnosed GDM, because their FPG before 24 WA was lower as compared with women without HG. Actually, hypothyroidism is associated with reduced hepatic gluconeogenesis and glycogen synthesis. FPG did not differ between two groups after 24 WA neithr at OGTT during their hospital stay.

It was hypothesized that, since HG women displayed higher HbA1c levels than those without, they could require an increased insulin dosage, or even one that was initiated at an earlier stage in the pregnancy. This was not the case. Additional **Supplementary Table 1** shows that the proportion of women needing insulin treatment was similar in the two groups. Insulin treatment was started later for women with HG probably because of late screening and subsequent DH. Only one study (31) evaluated the impact of HG on metabolic control in a GDM group of patients. The authors found that TSH was significantly associated with blood glucose levels and poor glycemic control but they did not provide treatment details.

We did not find any difference in terms of pregnancy outcomes, so the present exploratory results suggest that HG, when treated in some women, is not associated with adverse pregnancy outcomes. Nevertheless, we have to consider our results about pregnancy outcomes with caution as a number of women diagnosed with HG were treated with levothyroxine (our policy was to give levothyroxine in case of TSH ≥3 mUI/L after the first trimester, according to 2011 ATA recommendations). Indeed, treatment could have reset the metabolic differences between euthyroid and hypothyroid patients with GDM and have ameliorated pregnancy outcomes, masking HG adverse consequences. This is not consistent with the negative impact of HG in the first trimester, which has been shown to persist even after LT4 replacement (24, 32). The present study revealed that 9% of women with GDM exhibited HG. Assessing the prevalence of HG in women with GDM is also particularly challenging because the definitions and the indications for screening of both conditions have evolved throughout the years and vary worldwide. While several studies suggested that the prevalence of GDM could be increased in GH women (33–36), only few studies specifically assessed the prevalence of GH in GDM. A Pakistani group (31) found a prevalence of HG in GDM of 61.5% *vs*. 6%, *p* < 0.001, with 8.1% *vs*. 0% if only overt hypothyroidism is considered. This is unexpected, but it is a distinct population.

Vitacolonna et al. (37) did not find any difference in terms of TSH concentration or prevalence of HG in women with GDM. As in our study, the lack of data pertaining to the prevalence of HG in the non-GDM population constitutes a significant limitation in the interpretation of these findings.

Our study has several limitations. Firstly, this is a retrospective study. Secondly, as already mentioned, women with a TSH level ≥3 mUI/L after the first trimester were treated by levothyroxine replacement; thus, we could not draw conclusions about the role of HG on pregnancy outcomes in our GDM cohort. Thirdly, we did not have TSH levels in the first trimester; neither did we have fT4 levels at DH, but the increase in TSH in our population was mild (min–max: 4.01–13.83 mUI/L; median: 4.63 mUI/L; Q1, Q3: 4.25, 5.38 mUI/L) and overt hypothyroidism is not likely.

The strength of this study is that it shows that HG, known to be associated with an increased risk of GDM, may have a negative metabolic impact in the case of GDM, with TSH being associated with higher HbA1c and increased insulin resistance. Further studies are needed to prove the therapeutical implications of this metabolic profile.

## Data Availability

The raw data supporting the conclusions of this article will be made available by the authors, without undue reservation.
