# Supplementary material for: Association between hypothyroidism and metabolic profile in gestational diabetes mellitus
Source: Front Endocrinol (Lausanne). 2025 Sep 25;16:1614802. doi: 10.3389/fendo.2025.1614802 (PMC12507628; doi:10.3389/fendo.2025.1614802)
Supplement: Supplementary file 1 [file Table1.docx]

Supplemental Table 1: outcomes according to the presence of hypothyroidism during gestation. WA= week of amenorrhea.. LGA= large for gestational age. SGA= small for gestational age.

|  | Total (N=1290) | TSH [0.27-4.0] (N=1173) | TSH >4.0 (N=117) | p |
| --- | --- | --- | --- | --- |
| **Insulin Treatment during Pregnancy, n(%)** |  |  |  | 0.48 |
| no | 702 (54.4%) | 642 (54.7%) | 60 (51.3%) |  |
| yes | 588 (45.6%) | 531 (45.3%) | 57 (48.7%) |  |
| **WA Insulin Starting (weeks)**  **N= 516** | 29.54 (5.53) | 29.32 (5.55) | 31.82 (4.90) | 0.004 |
| **Delay between DH and Insulin Starting (weeks)**  **N= 516** | 2.12 (3.28) | 2.17 (3.36) | 1.61 (2.24) | 0.14 |
| **Total Insulin Daily Dose at Delivery**  **N= 493** | 25.56 (24.77) | 25.93 (25.15) | 21.39 (19.87) | 0.26 |
| **Pregnancy Weight Gain (kg)**  **N=1160** | 9.56 (5.44) | 9.53 (5.45) | 9.91 (5.41) | 0.50 |
| **Weight Outcomes** |  |  |  | 0.75 |
| LGA, n(%) | 172 (13.3%) | 158 (13.5%) | 14 (12.0%) |  |
| Normal, n(%) | 978 (75.8%) | 886 (75.5%) | 92 (78.6%) |  |
| SGA, n(%) | 140 (10.9%) | 129 (11.0%) | 11 (9.4%) |  |
| **Precampsia** |  |  |  | 0.836 |
| No | 1253 (97.1%) | 1139 (97.1%) | 114 (97.4%) |  |
| Yes | 37 (2.9%) | 34 (2.9%) | 3 (2.6%) |  |
| **Cesarian section, n(%)** |  |  |  | 0.179 |
| No | 939 (72.8%) | 860 (73.3%) | 79 (67.5%) |  |
| Yes | 351 (27.2%) | 313 (26.7%) | 38 (32.5%) |  |
| **Delivery Term (weeks)** | 39.48 (1.56) | 39.48 (1.56) | 39.53 (1.61) | 0.7426 |
| **Preterm delivery, n(%)** |  |  |  | 0.7940 |
| <37 WA | 81 (6.3%) | 73 (6.2%) | 8 (6.8%) |  |
| >=37 WA | 1209 (93.7%) | 1100 (93.8%) | 109 (93.2%) |  |
| **Offspring hospitalisation in NICU, n(%)**  N= 1024 |  |  |  | 0.2280 |
| No | 1024 (79.5%) | 936 (79.9%) | 88 (75.2%) |  |
| Yes | 264 (20.5%) | 235 (20.1%) | 29 (24.8%) |  |
| **Respiratory Distress, n(%)** |  |  |  | 0.7293 |
| No | 1232 (95.5%) | 1121 (95.6%) | 111 (94.9%) |  |
| Yes | 58 (4.5%) | 52 (4.4%) | 6 (5.1%) |  |
| **Fœtal/ Neonatal Death, n(%)** |  |  |  | 1.0000 |
| No | 1288 (99.8%) | 1171 (99.8%) | 117 (100.0%) |  |
| Yes | 2 (0.2%) | 2 (0.2%) | 0 (0.0%) |  |
| **Malformation, n(%)** |  |  |  | 0.4765 |
| No | 1263 (97.9%) | 1150 (98.0%) | 113 (96.6%) |  |
| Yes | 27 (2.1%) | 23 (2.0%) | 4 (3.4%) |  |
| **Neonatal hypoglycemia, n(%)** |  |  |  | 1.0000 |
| No | 1267 (98.2%) | 1152 (98.2%) | 115 (98.3%) |  |
| Yes | 23 (1.8%) | 21 (1.8%) | 2 (1.7%) |  |
| **Hyperbilirubinemia, n(%)** |  |  |  | 0.4598 |
| No | 1261 (97.8%) | 1145 (97.6%) | 116 (99.1%) |  |
| Yes | 29 (2.2%) | 28 (2.4%) | 1 (0.9%) |  |
